# Effect of ivermectin on scabies: a retrospective evaluation

**DOI:** 10.1186/s12879-025-11315-5

**Published:** 2025-07-23

**Authors:** Ömer Karakoyun, Erhan Ayhan, İsmail Yıldız

**Affiliations:** 1 Present Address: Department of Dermatology and Venereal Diseases, Dicle University Faculty of Medicine, Diyarbakir, Türkiye; 2 Department of Biostatistics, Dicle University Faculty of Medicine, Diyarbakir, Türkiye

**Keywords:** Ivermectin, Scabies, Treatment

## Abstract

**Objective:**

This study, aimed at determining the effect of ivermectin on scabies, which has recently reached epidemic proportions, was conducted by the Department of Dermatology and Venereology at Dicle University. The study aims to evaluate the success of ivermectin in the treatment of scabies, identify variables affecting this success, and contribute positively to the development of future national treatment protocols. Additionally, the study seeks to test the hypothesis that ivermectin, which is significantly easier to use in cases of failure with topical treatments, is a good first-line treatment option.

**Materials and methods:**

In this retrospective study, 412 patients diagnosed with scabies via clinical examination by a specialist physician and recommended a 200 µg/kg dose of ivermectin at one-week intervals, who presented to Dicle University Dermatology and Venereology Clinic between January 1, 2023, and June 30, 2024, were examined. Fifty-two patients whose records lacked the parameters evaluated in the study were excluded. A total of 360 patients were included in the study. Data on children under five years of age, those weighing less than 15 kg, and pregnant or lactating women were not obtained due to insufficient information regarding oral ivermectin use in these groups. Data were evaluated with SPSS-21.0 statistical program and the value, mean, median value, standard deviation, incidence rate and frequency of each parameter in total patients were recorded. Associations were analysed using Kolmogorov-Smirnov test, dependent t test, Wilcoxon test, Pearson Chi-square (χ2) test, Yates Chi-square (χ2) test, Fisher Chi-square (χ2) test analysis, Mc-Nemar test, Pearson/spearman correlation analysis, logistic regression analysis. A *p*-value of < 0.05 was considered statistically significant.

**Results:**

The ivermectin treatments for all 360 patients were prescribed by a specialist physician, and 78.6% (283) of the patients benefited from the treatment. Of these 360 patients, 295 (81.94%) had tried at least one other treatment option before ivermectin and did not benefit from it, while 66.1% (43 out of 65) of those who had not previously undergone treatment benefited from ivermectin. Furthermore, 81.36% (240 out of 295) of patients who did not respond to previous treatments benefited from ivermectin.

**Conclusion:**

This study concluded that ivermectin could be a significant treatment option for patients diagnosed with scabies. The superiority of appropriately dosed ivermectin treatment over other treatments was observed, particularly in patients resistant to other scabies treatments.

## Introduction

Scabies is a highly contagious dermatological disease caused by the ectoparasite Sarcoptes scabiei, which burrows into specific layers of the skin and causes severe itching. It is more frequently observed during the autumn and winter months and affects individuals of all ages and social strata [1, 2].

Studies in recent years have shown that the incidence of scabies is increasing worldwide, including in our country [3, 4].

Recent literature also emphasizes that this increase persists and that preventive measures are necessary [5–7].

Topical and systemic treatments, either alone or in combination, are used to kill the mites and their eggs responsible for scabies [8, 9]. However, the failure rates of topical treatments, particularly permethrin, have been increasing in recent years [10, 11]. This failure has been attributed to various factors, primarily resistance [12, 13].

Among these treatment options, ivermectin—a potent antiparasitic drug with multiple indications, including pediculosis, rosacea, and Demodex infestation—has emerged as a prominent option. It is recommended at a dose of 200 µg/kg administered twice at a one-week interval for scabies [14, 15].

Many studies have compared ivermectin with other scabies treatment options.

While permethrin is more commonly preferred in scabies treatment, ivermectin has been found to be more effective, particularly in crusted scabies, HIV-positive patients, bedridden patients, and epidemic cases [16–18].

Additionally, four comparative studies have found that one to two doses of oral ivermectin at 200 µg/kg are as effective as topical treatments such as benzyl benzoate, lindane, and permethrin, although contradictory findings have also been reported in other studies [19–21]. The study aims to evaluate the success of ivermectin in the treatment of scabies, to identify the variables affecting this success and to contribute positively to the development of future national treatment protocols, as well as to test the hypothesis that ivermectin, which is significantly easier to use in cases of failure with topical treatments, is a good first-line treatment.

## Materials and methods

In this retrospective study, 412 patients diagnosed with scabies via clinical examination by a specialist physician and recommended a 200 µg/kg dose of ivermectin at one-week intervals, who presented to Dicle University Dermatology and Venereology Clinic between January 1, 2023, and June 30, 2024, were examined. Fifty-two patients whose records lacked the parameters evaluated in the study were excluded. A total of 360 patients were included in the study. Data on children under five years of age, those weighing less than 15 kg, and pregnant or lactating women were not obtained due to insufficient information regarding oral ivermectin use in these groups. Data were evaluated with SPSS-21.0 statistical program and the value, mean, median value, standard deviation, incidence rate and frequency of each parameter in total patients were recorded. Associations were analysed using Kolmogorov-Smirnov test, dependent t test, Wilcoxon test, Pearson Chi-square (χ2) test, Yates Chi-square (χ2) test, Fisher Chi-square (χ2) test analysis, Mc-Nemar test, Pearson/spearman correlation analysis, logistic regression analysis. A *p*-value of < 0.05 was considered statistically significant.

## Results

**Fig. 1:**
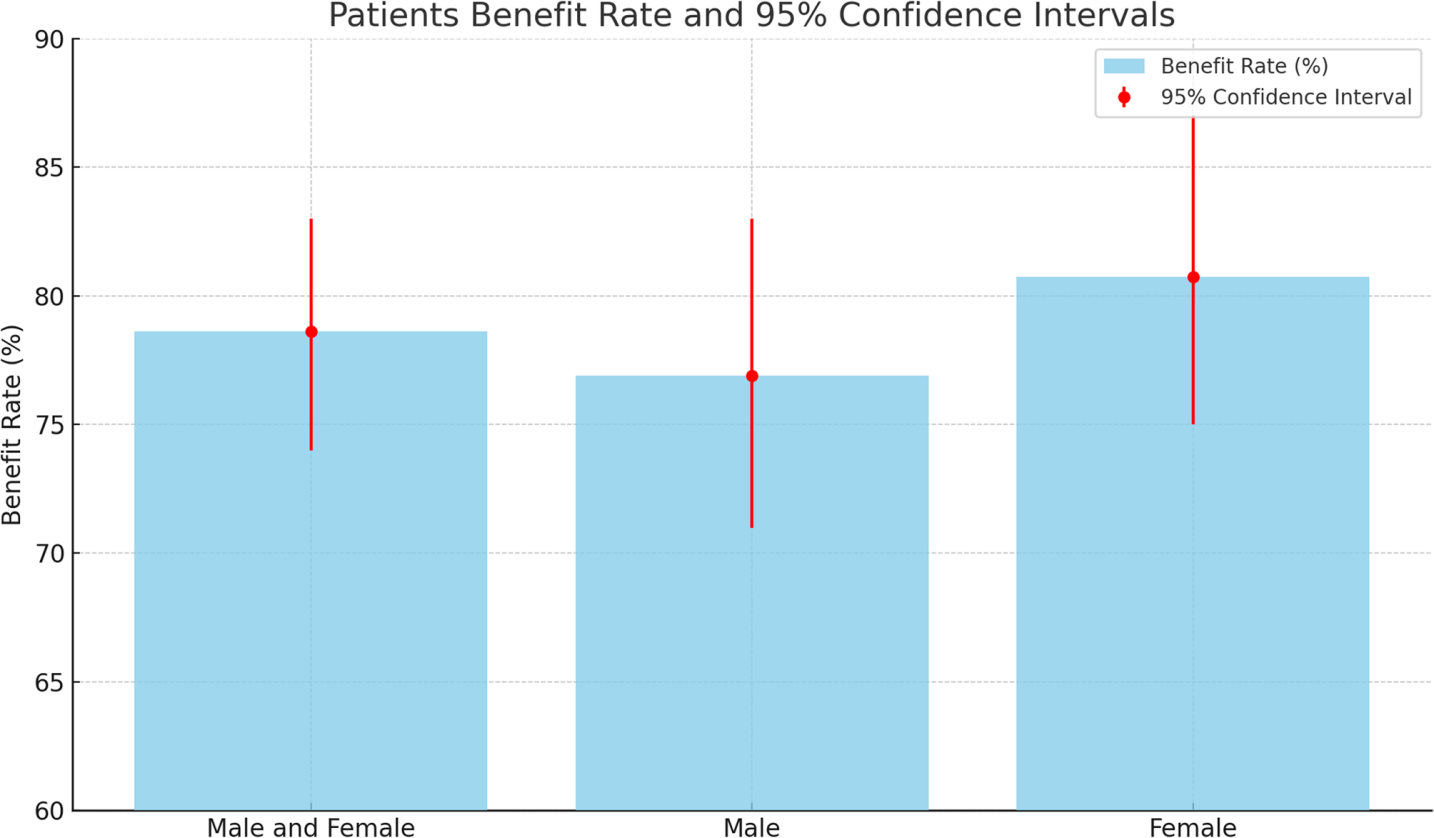
Patients benefit rate and 95% confidence intervals

Of the 360 patients, 44.7% (161) were female, and 55.3% (199) were male (Table 1). Similar complaints were present in the families of 76.1% (274) of the total patients. Figure 1 shows the rate of benefit from treatment according to gender.All 360 patients were treated with ivermectin by a specialist physician, and 78.6% (283) of the patients benefited from the treatment. During treatment, all family members of 232 patients (64.4%) received treatment, while in 57 patients (15.8%), some family members received treatment while others did not. In 65 patients (18.1%), no family members other than the patient received treatment. The treatment status of the family was unclear in six patients (1.7%). A significant relationship was found between whether the patient benefited from ivermectin and whether the treatment was administered across the entire family (*p* < 0.0001). A total of 97.8% (352) of the patients received the medication in the recommended two doses. A significant correlation was also found between the response to ivermectin and the administration of the two doses (*p* < 0.0001). It was determined that pruritus was alleviated or resolved in 281 out of the 283 patients who benefited from the medication. Pruritus resolved within 10 days in 50 patients (17.8%), within 10–20 days in 132 patients (47%), and after more than 20 days in 99 patients (35.2%). No significant relationship was observed between the duration of pruritus resolution and whether the entire family received treatment (*p* = 0.223). However, a significant relationship was found between this duration and the total number of individuals living in the household and the number of individuals experiencing pruritus (*p* = 0.0006). A significant relationship was also observed between this duration and the number of rooms in the household (*p* = 0.0006). Excluding 65 patients (18.06%), 295 patients (81.94%) had tried at least one other treatment option before ivermectin and had not benefited from it. Of the 65 patients who had not received any prior treatment, 43 (66.1%) benefited from ivermectin, while 240 out of the 295 patients (81.36%) who had not responded to previous treatments also benefited from ivermectin ).

**Table 1:** General demographic information and response to treatment

Gender	Number of Patients	Average Age	Benefit (%)	95% Confidence Interval
Male and Female	360(%100)	28.91	78.61	0.74–0.83
Male	199(%55,3)	28.88	76.88	0.71–0.83
Female	161(%44,7’)	28.95	80.74	0.75–0.87

## Discussion

As previously mentioned, ivermectin is a broad-spectrum antiparasitic agent that can be used both topically and systemically for various dermatological conditions, such as pediculosis, rosacea, Demodex infestation, and scabies. Its use has recently increased [14, 15].

While the incidence of scabies has been reported to rise both nationally and globally, numerous studies have been conducted on the reliability and efficacy of treatment options [5–7].

The number of patients diagnosed and treated for scabies but returning without a response to treatment is increasing daily. This situation has prompted the investigation of new treatment options and the development of new treatment protocols to address scabies at a national level and control its spread. Systemic ivermectin, one of these treatment options, has recently been increasingly utilized for scabies, which has reached epidemic levels in Türkiye, thanks to a program funded by the Ministry of Health of the Republic of Türkiye. This program has provided an opportunity to study the safety and efficacy of the drug. To our knowledge, this study is one of the most extensive investigations into the use and efficacy of systemic ivermectin for scabies.

Data obtained in this study address the contagious nature of scabies, the efficacy of systemic ivermectin, its superiority in patient groups resistant to other treatments, and its effectiveness when taken in the recommended dose and when other individuals in shared living spaces are also treated. The data obtained in this study are generally consistent with those in the literature, though some findings align with opposing studies.

This study also demonstrated that ivermectin treatment is a significant option for scabies patients unresponsive to other treatments. Of the 295 patients unresponsive to prior treatments, 240 (81.36%) benefited from ivermectin.

**Fig. 2:**
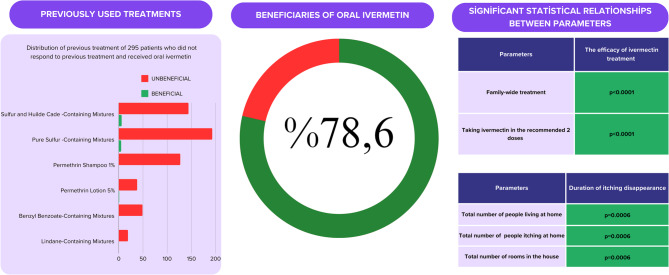
General data obtained in the study and statistically significantly related parameters

As shown in Fig. 2, the previous treatments of the 295 patients who were unresponsive were entirely topical. The challenges of using topical treatments and the presence of similar complaints among other individuals in shared living spaces in 256 of these patients may be the primary reasons for the failure of previous treatments. Regarding the difficulty of use, patients reported hesitations about applying sulfur-containing mixtures due to their unpleasant odors, which highlights that noncompliance with the application method contributed to treatment unresponsiveness.

As also noted in the literature, incorrect application is one of the significant reasons for treatment failure [16, 17]. Furthermore, as shown in Figs. 2 and 12 patients benefited from some topical treatment options but were subsequently reinfected. The medical history of these 12 patients revealed that they traveled to another location from their shared living space after treatment and experienced a recurrence of symptoms and lesions upon their return. This emphasizes, consistent with many other studies, that all individuals in shared living spaces, regardless of whether they are symptomatic, should receive treatment [18, 19, 20]. The study also found that 219 (74.23%) of the 295 patients unresponsive to topical treatments who used oral ivermectin underwent family-wide treatment, while 207 (86.24%) of the 240 patients who benefited from ivermectin also received family-wide treatment.

In line with the study’s findings, environmental control has been emphasized as an additional important factor contributing to treatment success, along with the two main factors identified [14].

The study also showed that other family members should also receive treatment in the same period with a positive response to ivermectin treatment (*p* < 0.0001).

In order to benefit from ivermectin, it was determined that it should be taken in two doses as recommended in other publications in the literature with a significant relationship in line with our data (*p* < 0.0001).

Particularly, Riccardo Balestri and colleagues’ dose comparison study on oral ivermectin in scabies treatment demonstrated the superiority of two doses, with a response rate of 98% compared to 58% for one dose [21]. Several current guidelines, such as the European or UK National guidelines, already recommend two rounds of oral ivermectin treatment [22].

**Fig. 3:**
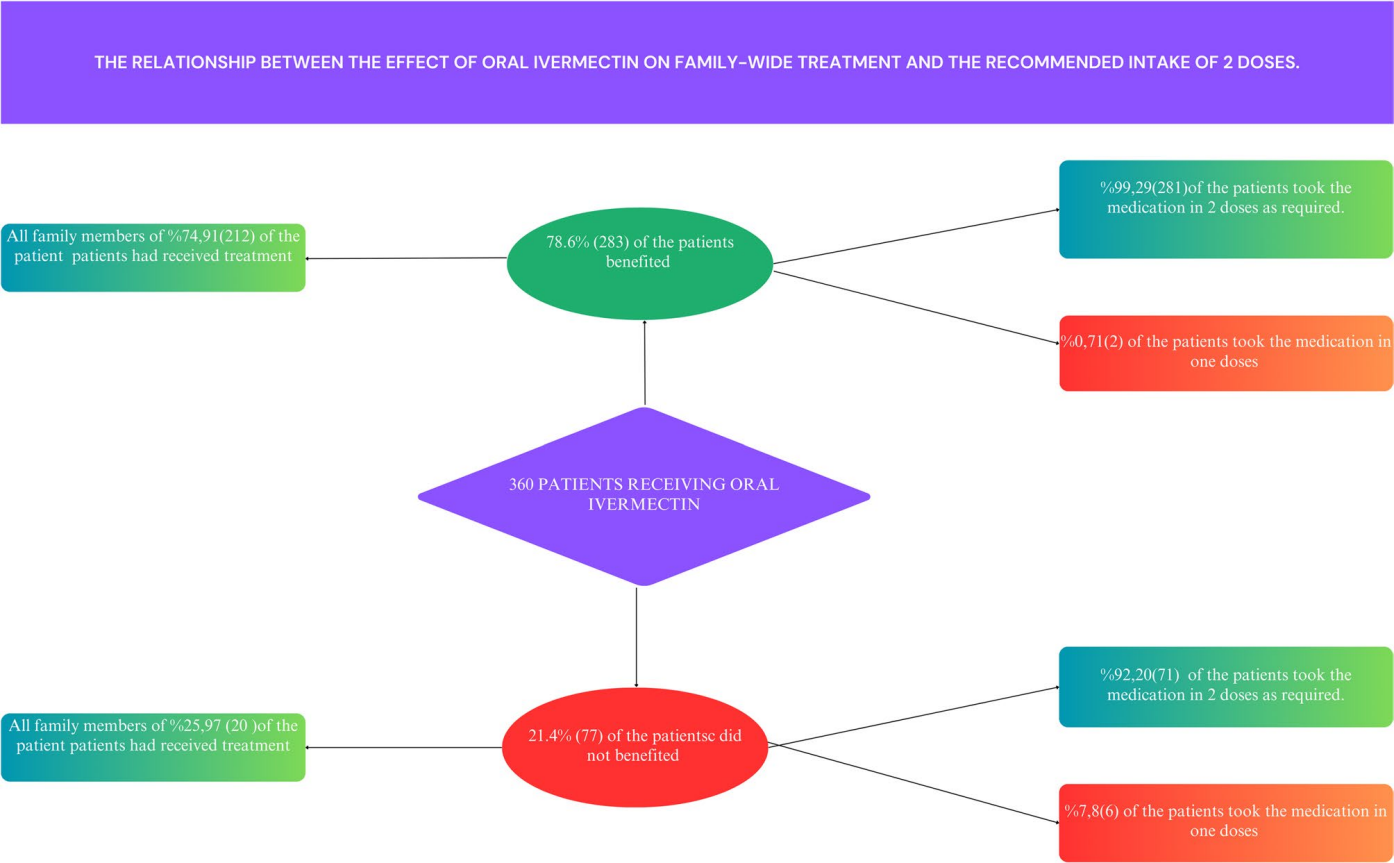
The relationship between the effect of oral ivermectin on family-wide treatment and the recommended intake of 2 doses

As shown in Fig. 3, the importance of family-wide treatment and administering oral ivermectin in the recommended two doses is evident.

Unlike other studies in the literature, this study found oral ivermectin to be superior in patient groups unresponsive to other treatment options, such as topical permethrin, benzyl benzoate, lindane, and sulfur-containing mixtures [23–25].

A Cochrane review conducted in 2007 also emphasized the superiority of oral ivermectin over benzyl benzoate [26]. This study supports the Cochrane review, as 49 of the 240 patients who responded to oral ivermectin had not benefited from benzyl benzoate-containing mixtures.

A study by Bachewar NP and colleagues in 2009 confirmed the superiority of oral ivermectin over benzyl benzoate and recommended oral ivermectin for patients unresponsive to benzyl benzoate [27]. The data from this study support this recommendation.

Another Cochrane review in 2018 found the effects of permethrin and oral ivermectin to be similar despite poor reporting as a significant limitation [28].

A study by Manjhi PK and colleagues yielded similar results [29].

In 2020, Behera P. and colleagues conducted a cluster randomized controlled trial involving 2,751 participants, which found oral ivermectin to be superior to topical permethrin [30]. Similarly, a study conducted in Fiji in 2015 with 2,051 participants also demonstrated the superiority of oral ivermectin [31]. A common feature of these two studies, besides highlighting the superiority of oral ivermectin, is that they were conducted in endemic regions. Perhaps oral ivermectin should be recommended as the first-line treatment in endemic areas.

According to a systematic review and meta-analysis conducted by Mbuagbaw L. and colleagues in 2023, treatment failure rates with two doses of oral ivermectin were significantly lower than those with topical permethrin. Consistent with our study, this analysis suggested that two doses of ivermectin are more effective [32].

Balestri R. and colleagues, in a study including patients unresponsive to topical permethrin, similarly found oral ivermectin to be superior [33].

While there are studies in the literature suggesting the superiority of permethrin over oral ivermectin, causing some confusion, large-scale meta-analyses provide evidence supporting the superiority of oral ivermectin over topical permethrin.

In 99%, 3% of patients who benefited from ivermectin, pruritus did not immediately cease but subsided gradually, ending with relief. A significant relationship was observed between the duration of this process (not reported in other studies in the literature) and the total number of household members, the number of individuals experiencing pruritus, and the number of rooms in the shared living space (*p* = 0.0006).

No serious adverse events were observed that would prevent the administration of the second dose of oral ivermectin or following its administration during treatment. Among 360 patients, only seven (1.91%) experienced adverse events potentially attributable to the medication. Of these, two reported increased pruritus during the first month of treatment, two experienced nausea and vomiting, two had diarrhea, and one reported abdominal pain. Consistent with the literature, these findings suggest that ivermectin is a safe drug with high tolerability [34].

### Limitations

This study was conducted retrospectively. The primary limitation is that most of the data were based on patient-reported histories obtained by clinicians, and the retrospective design of the study posed constraints. Additionally, since the study relied on the review of patient files not initially designed for data collection, some missing information was inevitable. In order to contribute to the literature, a control group can be added to our study, and comparisons can be made.

## Conclusion

In conclusion, ivermectin treatment was found to be an effective choice, particularly in patients unresponsive to other treatments. It was also observed that administering two doses is essential for achieving therapeutic benefits, and other individuals in shared living spaces must also be treated during the treatment period. Patients should be informed that even though lesions may heal following ivermectin treatment, pruritus may persist for a certain period.

## Data Availability

The data that support the findings of this study are available from the Dicle University Faculty of Medicine, but restrictions apply to the availability of these data, which were used under license for the current study, and so are not publicly available. Data are available, however, from the corresponding author upon reasonable request and with permission of the Dicle University Faculty of Medicine.
